# Risk stratification of childhood infection using host markers of immune and endothelial activation in Asia (Spot Sepsis): a multi-country, prospective, cohort study

**DOI:** 10.1016/S2352-4642(25)00183-X

**Published:** 2025-09-01

**Authors:** Arjun Chandna, Constantinos Koshiaris, Raman Mahajan, Riris Adono Ahmad, Dinh Thi Van Anh, Khalid Shams Choudhury, Suy Keang, Phung Nguyen The Nguyen, Sayaphet Rattanavong, Souphaphone Vannachone, Spot Sepsis Investigator Group, Mikhael Yosia, Naomi Waithira, Mohammad Yazid Abdad, Janjira Thaipadungpanit, Paul Turner, Phan Huu Phuc, Dinesh Mondal, Mayfong Mayxay, Bui Thanh Liem, Elizabeth A Ashley, Eggi Arguni, Rafael Perera-Salazar, Melissa Richard-Greenblatt, Yoel Lubell, Sakib Burza

**Affiliations:** Cambodia Oxford Medical Research Unit, Angkor Hospital for Children, Siem Reap, Cambodia; Centre for Tropical Medicine and Global Health, University of Oxford, Oxford, UK; Department of Primary Care Health Sciences, University of Oxford, Oxford, UK; Department of Primary Care and Population Health, University of Nicosia Medical School, Nicosia, Cyprus; Médecins Sans Frontières, Spain; Centre for Tropical Medicine, Universitas Gadjah Mada, Yogyakarta, Indonesia; Viet Nam National Children’s Hospital, Ha Noi, Viet Nam; Médecins Sans Frontières, Spain; Cambodia Oxford Medical Research Unit, Angkor Hospital for Children, Siem Reap, Cambodia; Paediatric Intensive Care Department, Angkor Hospital for Children, Siem Reap, Cambodia; University of Medicine and Pharmacy at Ho Chi Minh City, Ho Chi Minh City, Viet Nam; Lao-Oxford-Mahosot Hospital-Wellcome Trust Research Unit, Mahosot Hospital, Vientiane, Laos; Lao-Oxford-Mahosot Hospital-Wellcome Trust Research Unit, Mahosot Hospital, Vientiane, Laos; Médecins Sans Frontières, Spain; Centre for Tropical Medicine and Global Health, University of Oxford, Oxford, UK; Mahidol Oxford Tropical Medicine Research Unit, Mahidol University, Bangkok, Thailand; Centre for Tropical Medicine and Global Health, University of Oxford, Oxford, UK; Mahidol Oxford Tropical Medicine Research Unit, Mahidol University, Bangkok, Thailand; Mahidol Oxford Tropical Medicine Research Unit, Mahidol University, Bangkok, Thailand; Faculty of Tropical Medicine, Mahidol University, Bangkok, Thailand; Cambodia Oxford Medical Research Unit, Angkor Hospital for Children, Siem Reap, Cambodia; Centre for Tropical Medicine and Global Health, University of Oxford, Oxford, UK; Viet Nam National Children’s Hospital, Ha Noi, Viet Nam; International Centre for Diarrhoeal Disease Research, Dhaka, Bangladesh; Centre for Tropical Medicine and Global Health, University of Oxford, Oxford, UK; Lao-Oxford-Mahosot Hospital-Wellcome Trust Research Unit, Mahosot Hospital, Vientiane, Laos; Institute for Research and Education Development, University of Health Sciences, Vientiane, Laos; University of Medicine and Pharmacy at Ho Chi Minh City, Ho Chi Minh City, Viet Nam; Centre for Tropical Medicine and Global Health, University of Oxford, Oxford, UK; Lao-Oxford-Mahosot Hospital-Wellcome Trust Research Unit, Mahosot Hospital, Vientiane, Laos; Centre for Tropical Medicine, Universitas Gadjah Mada, Yogyakarta, Indonesia; Department of Primary Care Health Sciences, University of Oxford, Oxford, UK; Centre for Tropical Medicine and Global Health, University of Oxford, Oxford, UK; Department of Laboratory Medicine and Pathobiology, University of Toronto, Toronto, ON, Canada; Hospital for Sick Children, Toronto, ON, Canada; Centre for Tropical Medicine and Global Health, University of Oxford, Oxford, UK; Mahidol Oxford Tropical Medicine Research Unit, Mahidol University, Bangkok, Thailand; Amsterdam Institute of Global Health and Development, Amsterdam, Netherlands; Médecins Sans Frontières, Spain; Clinical Research Department, London School of Hygiene and Tropical Medicine, London, UK; Health in Harmony, Portland, OR, USA

## Abstract

**Background:**

Prognostic tools for febrile illnesses are urgently required in resource-constrained community contexts. Circulating immune and endothelial activation markers stratify risk in common childhood infections. We aimed to assess their use in children with febrile illness presenting from rural communities across Asia.

**Methods:**

Spot Sepsis was a prospective cohort study across seven hospitals in Bangladesh, Cambodia, Indonesia, Laos, and Viet Nam that serve as a first point of contact with the formal health-care system for rural populations. Children were eligible if aged 1–59 months and presenting with a community-acquired acute febrile illness that had lasted no more than 14 days. Clinical parameters were recorded and biomarker concentrations measured at presentation. The primary outcome measure was severe febrile illness (death or receipt of organ support) within 2 days of enrolment. Weighted area under the receiver operating characteristic curves (AUC) were used to compare prognostic accuracy of endothelial activation markers (ANG-1, ANG-2, and soluble FLT-1), immune activation markers (CHI3L1, CRP, IP-10, IL-1ra, IL-6, IL-8, IL-10, PCT, soluble TNF-R1, soluble TREM1 [sTREM1], and soluble uPAR), WHO danger signs, the Liverpool quick Sequential Organ Failure Assessment (LqSOFA) score, and the systemic inflammatory response syndrome (SIRS) score. Prognostic accuracy of combining WHO danger signs and the best performing biomarker was analysed in a weighted logistic regression model. Weighted measures of classification were used to compare prognostic accuracies of WHO danger signs and the best performing biomarker and to determine the number of children needed to test (NNT) to identify one additional child who would progress to severe febrile illness. The study was prospectively registered on ClinicalTrials.gov, NCT04285021.

**Findings:**

3423 participants were recruited between March 5, 2020, and Nov 4, 2022, 18 (0·5%) of whom were lost to follow-up. 133 (3·9%) of 3405 participants developed severe febrile illness (22 deaths, 111 received organ support; weighted prevalence 0·34% [95% CI 0·28–0·41]). sTREM1 showed the highest prognostic accuracy to identify patients who would progress to severe febrile illness (AUC 0·86 [95% CI 0·82–0·90]), outperforming WHO danger signs (0·75 [0·71–0·80]; p<0·0001), LqSOFA (0·74 [0·69–0·78]; p<0·0001), and SIRS (0·63 [0·58–0·68]; p<0·0001). Combining WHO danger signs with sTREM1 (0·88 [95% CI 0·85–0·91]) did not improve accuracy in identifying progression to severe febrile illness over sTREM1 alone (p=0·24). Sensitivity for identifying progression to severe febrile illness was greater for sTREM1 (0·80 [95% CI 0·73–0·85]) than for WHO danger signs (0·72 [0·66–0·79]; NNT=3000), whereas specificities were comparable (0·81 [0·78–0·83] for sTREM1 *vs* 0·79 [0·76–0·82] for WHO danger signs). Discrimination of immune and endothelial activation markers was best for children who progressed to meet the outcome more than 48 h after enrolment (sTREM1: AUC 0·94 [95% CI 0·89–0·98]).

**Interpretation:**

sTREM1 showed the best prognostic accuracy to discriminate children who would progress to severe febrile illness. In resource-constrained community settings, an sTREM1-based triage strategy might enhance early recognition of risk of poor outcomes in children presenting with febrile illness.

## Introduction

Whether to refer a child with febrile illness to hospital is a challenging decision facing front-line community health-care workers globally, particularly in resource-limited and conflict-affected settings.^1^ Each day, children who will develop severe disease are missed, while referrals of illnesses suitable for community-based management incur avoidable cost for caregivers and health systems.^2,3^ In rural locations of many low-income and middle-income countries, referral decisions are complex and influenced by poorly functioning health systems, limited referral infrastructure, and geographical, climatic, socioeconomic, and cultural factors.^4,5^

In under-resourced peripheral health-care settings, WHO recommends general danger signs (convulsions, intractable vomiting, lethargy, or prostration) to identify children with febrile illness requiring hospital referral.^6,7^ Yet, these danger signs suffer from considerable inter-observer variability and lack both sensitivity and specificity.^8,9^ Absence of data on children managed in the community setting renders their validity questionable.^10^ Better risk stratification tools for common childhood infections are needed.

Circulating markers of immune and endothelial activation have consistently demonstrated ability to risk stratify paediatric fever syndromes agnostic to pathogen aetiology.^11,12^ Increased concentrations of these markers indicate loss of endothelial integrity and microvascular quiescence that contribute to disease progression, organ dysfunction, and death.^13,14^ They could be of particular value for identifying patients whose illness severity is not clinically apparent at presentation and who might be discharged and deteriorate at home.^15^ Whether these findings could be applied at the community setting in Asia is unknown. Most evidence stems from single-site studies of children who had been admitted to hospital in locations where prevalent causes of infection and host susceptibility patterns differ.^11,16,17^

Spot Sepsis was a multi-country study of markers of immune and endothelial activation in children presenting with community-acquired acute febrile illnesses, with two main objectives: to examine the prognostic performance of individual markers of immune and endothelial activation (reported herein); and to develop a clinical prediction model (ongoing, to be reported separately).^18^ Study sites and participants were purposefully selected to be representative of the intended use-case in peripheral health-care settings. We aimed to determine whether presenting concentrations of markers of immune and endothelial activation predict disease progression, thereby assessing their potential to identify children at risk of severe disease, relative to currently used clinical tools.

## Methods

### Study design and participants

Spot Sepsis was a prospective cohort study with seven participating hospitals in Bangladesh, Cambodia, Indonesia, Laos, and Viet Nam that served as first points of contact with the formal health-care system for rural populations (appendix 3 pp 4–5). Recruitment commenced on March 5, 2020, and continued until Nov 4, 2022.

Children aged 1–59 months presenting with acute febrile illnesses (axillary temperature ≥37·5°C or <35·5°C or a history of fever in the preceding 24 h) lasting no longer than 14 days were eligible for inclusion.^6,19^ Exclusion criteria were previous admission to any health facility during the current illness episode, receipt of more than 15 min parenteral treatment before screening (supplemental oxygen, intravenous fluids, or intravenous, intramuscular or nebulised medication),^20^ presentation within 3 days of routine immunisations, trauma as the reason for attendance, or specific known comorbidities (chronic infection, immunosuppression, or active [symptomatic or medicated] cardiorespiratory conditions). Participants could only be enrolled once.

Patients were screened during daytime working hours. Screening was stratified by admission status. Admissions could occur via the emergency or outpatient departments. Inpatients were screened consecutively upon arrival at the emergency department or inpatient ward. Patients admitted from the outpatient department typically did not receive treatment before arrival on the inpatient ward. Patients attending the emergency department typically received treatment promptly and were either admitted or kept for a period of observation. Thus, all patients presenting to the emergency department (those admitted and those kept for a period of observation) were considered inpatients.

Because of high outpatient numbers, outpatient screening was randomised. Outpatients to be approached were randomly allocated by computer-generated random number tables, with the preceding week’s routinely collected hospital attendance data providing the sampling frame. Screening of outpatients occurred before assessment by the clinical team. On rare occasions when the health worker decided to admit a patient just recruited into the outpatient strata, the participant was transferred to the inpatient strata and the next patient specified on the random number table was approached for screening.

Variables for data collection were informed by systematic review of the literature.^21^ Prioritisation and standardisation followed guidance set out by the Pediatric Sepsis Predictors Standardization working group.^22^

Biomarkers were identified following review of the literature (appendix 3 p 6). Biomarkers useful for risk stratification in primary care, where the cause of infection is typically unknown at the time of assessment, must be predictive across a range of pathogens. Hence, biomarkers with mechanistic links to final common pathways of severe febrile illness and sepsis were prioritised.^13,23,24^ Accessibility of point-of-care tests for the biomarkers in resource-constrained community contexts was considered, although absence of a test did not preclude a biomarker from being included.

Markers of endothelial activation included ANG-1, ANG-2, and soluble FLT-1 (sFLT-1; also known as sVEGFR-1). Markers of immune activation included CHI3L1, CRP, IP-10 (also known as CXCL10), IL-1ra, IL-6, IL-8, IL-10, PCT, soluble TNF-R1 (sTNF-R1; also known as TNFRSF1A), soluble TREM1 (sTREM1), and soluble uPAR (suPAR). Lactate, glucose, and haemoglobin were included because they are measurable using inexpensive rapid tests, are familiar to many clinicians, have prognostic value,^21^ and are promoted in paediatric sepsis guidelines.^19,25,26^

The Liverpool quick Sequential Organ Failure Assessment (LqSOFA; measuring mental state, capillary refill time, age-adjusted respiratory rate, and age-adjusted heart rate) and the systemic inflammatory response syndrome (SIRS; measuring age-adjusted respiratory rate, age-adjusted heart rate, temperature, and age-adjusted leukocyte count) scores were selected as additional comparators, alongside WHO danger signs (appendix 3 p 7).^27–30^

Caregivers of all participants provided informed written consent. The study was prospectively registered on ClinicalTrials.gov (NCT04285021) and received ethical approval from the sponsors and ethical review boards in all participating countries (appendix 3 p 8).

### Procedures

Trained study personnel measured vital signs and anthropometrics, assessed clinical signs (including WHO danger signs), and collected venous blood samples and nasopharyngeal swabs at enrolment (appendix 3 p 9). Clinical data were entered onto electronic case record forms using Android tablets via Open Data Kit Collect software. Venous blood samples and nasopharyngeal swabs were processed immediately. Complete blood counts were performed on site. Peripheral blood cultures were processed at accredited in-country laboratories. Aliquots of whole blood, EDTA-plasma, fluoride-oxalate-plasma, and universal transport medium were stored at –20°C or below. Samples were then transported at –80°C to the Mahidol Oxford Tropical Medicine Research Unit (MORU) laboratories in Bangkok, Thailand, for further analysis and biobanking.

Participants were followed up on days 2 and 28 after enrolment, with additional follow-up on day 1 and at discharge for inpatients. Follow-up was conducted in person if the participant remained at the study site or via telephone if they had been discharged. Participants were provided with routine care by their treating clinician. Given the higher level of training of health workers practising at the study sites (compared with community health-care providers), presence or absence of WHO danger signs are typically not the major drivers of patient management decisions. When feasible, the study supported collection and processing of peripheral blood cultures at the discretion of the clinical team. Study monitoring was conducted by the Clinical Trials Support Group at MORU in Bangkok, Thailand.

Biomarker concentrations were quantified in EDTA-plasma using the Simple Plex Ella microfluidic platform (ProteinSimple, San Jose, CA, USA) and suPARnostic ELISA (ViroGates, Copenhagen, Denmark), as described in appendix 3 (p 10). Lactate (LACT2, Roche Diagnostics, Mannheim, Germany) and glucose (GLUC3, Roche Diagnostics) concentrations were quantified in fluoride–oxalate plasma.

Nucleic acid was extracted from whole blood using the MagNA Pure 24 instrument and Total NA Isolation Kit (Roche Diagnostics, Indianapolis, IN, USA). Whole blood viral targets (chikungunya, dengue, Japanese encephalitis, and Zika virus) and bacterial targets (*Leptospira* spp, *Orientia tsutsugamushi*, and *Rickettsia* spp) were detected using laboratory-developed real-time PCR (rtPCR) multiplex assays. Respiratory pathogen targets were detected directly from nasopharyngeal swabs using the FilmArray RP2 panel (BioFire Diagnostics, Salt Lake City, UT, USA), with the exception of Cambodian samples. Cambodian respiratory samples were processed for influenza A and influenza B viruses and respiratory syncytial virus (RSV) using the FTD FLU/HRSV assay (Siemens, Erlangen, Germany). Samples from all sites were tested using an in-house developed multiplex rtPCR assay for the detection of SARS-CoV-2 from nasopharyngeal swabs based on the *E* and *N* genes, as described previously.^31^ Molecular targets were restricted to pathogens for which illness causality can be more confidently ascribed.^32^

### Outcomes

The primary outcome measure was development of severe febrile illness within 2 days of enrolment, defined as organ support (mechanical ventilation, non-invasive ventilation, inotropic therapy, or renal replacement therapy) or death.

### Statistical analysis

Given delays caused by the COVID-19 pandemic, the approach to sample size became pragmatic, with recruitment continuing for as long as resources permitted. The methods of Riley and colleagues^33^ were followed to estimate the number of parameters that could be included to build the clinical prediction model (work ongoing, to be reported separately), while minimising the risk of overfitting. Using an anticipated outcome prevalence of 1%, conservative *R*^2^ Nagelkerke of 0·15, and shrinkage factor of 0·9, it was estimated that more than 15 parameters could be used to derive the prediction model, confirming that the sample size was adequate for the univariate analyses presented here.

Complete case analyses were used as few data were missing among the primary comparators (immune and endothelial activation markers and WHO danger signs). Site-specific probability weights were applied to adjust for unequal probabilities of selection in the sample, arising due to random sampling of outpatients (appendix 3 pp 11–12), to ensure that the study population was representative of all eligible children presenting to the hospital and that the outcome prevalence reflected that which might be observed in community care settings.^34,35^ Categorical and continuous variables were summarised and compared using descriptive statistics and statistical comparisons made using the Wilcoxon rank sum, Pearson’s χ^2^, and Fisher’s exact test as appropriate.

The prognostic accuracy of each biomarker, WHO danger signs, and clinical severity scores was quantified using the weighted area under the receiver operating characteristic curve (AUC). When evaluating the combination of WHO danger signs and a biomarker, a weighted logistic regression model was used to generate predicted probabilities, which were subsequently used to estimate the AUC.^36^ Weighted measures of classification (sensitivity and specificity) were used to compare prognostic accuracies of WHO danger signs and the best performing biomarker and to determine the number of children needed to test (NNT) to identify one additional child who would progress to severe febrile illness.

Prespecified subgroup analyses included children with microbiologically confirmed infections and different presenting clinical syndromes. Prognostic accuracy was explored across prediction horizons (<4 h, ≥4 h, ≥24 h, and ≥48 h). These secondary analyses were planned to test the hypotheses that immune and endothelial activation markers were pathogen agnostic and would predict disease progression across different microbial aetiologies, and that value of biomarker measurements would be greatest in children whose illnesses progressed later after the point of presentation.^15,23,24^

### Role of the funding source

Médecins Sans Frontières, Spain, maintained a sponsor-investigator role in which it contributed to study design, data collection, data analysis, data interpretation, writing of the report, and decision to submit for publication. Wellcome had no role in study design, data collection, analysis, or interpretation, writing of the report, or decision to submit for publication.

## Results

**Figure 1:**
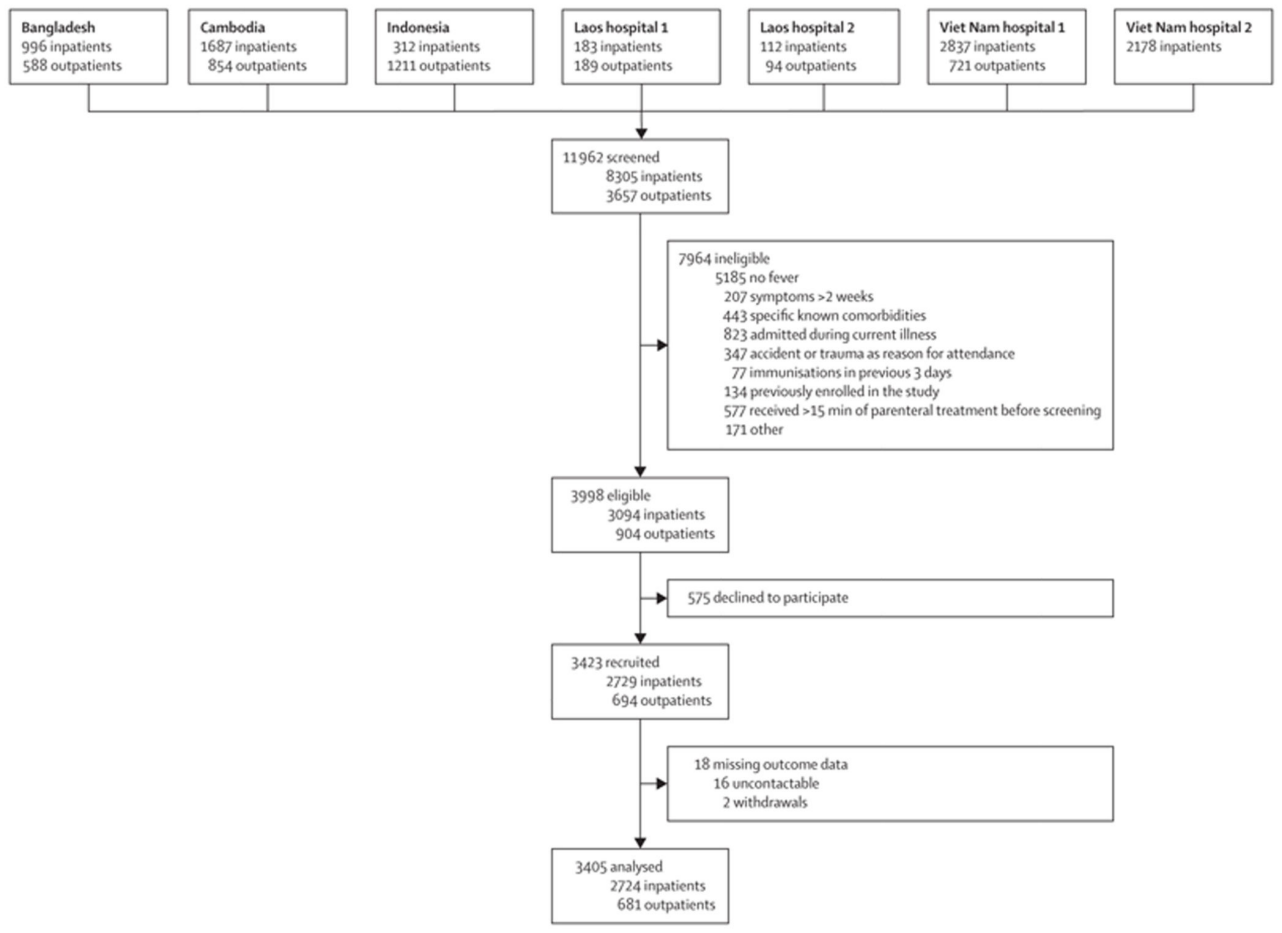
Study flowchart One reason for ineligibility is provided per patient, according to the hierarchy listed in the figure. In total, 1245 (15·6%) of 7964 ineligible children had more than one reason for ineligibility.

Between March 5, 2020, and Nov 4, 2022, 11 962 patients were screened, of whom 3998 (33·4%) were eligible and 3423 were recruited (575 [14·4%] declined to participate). 18 (0·5%) of 3423 recruited participants were lost to follow-up and excluded from further analyses (figure 1).

**Figure 2:**
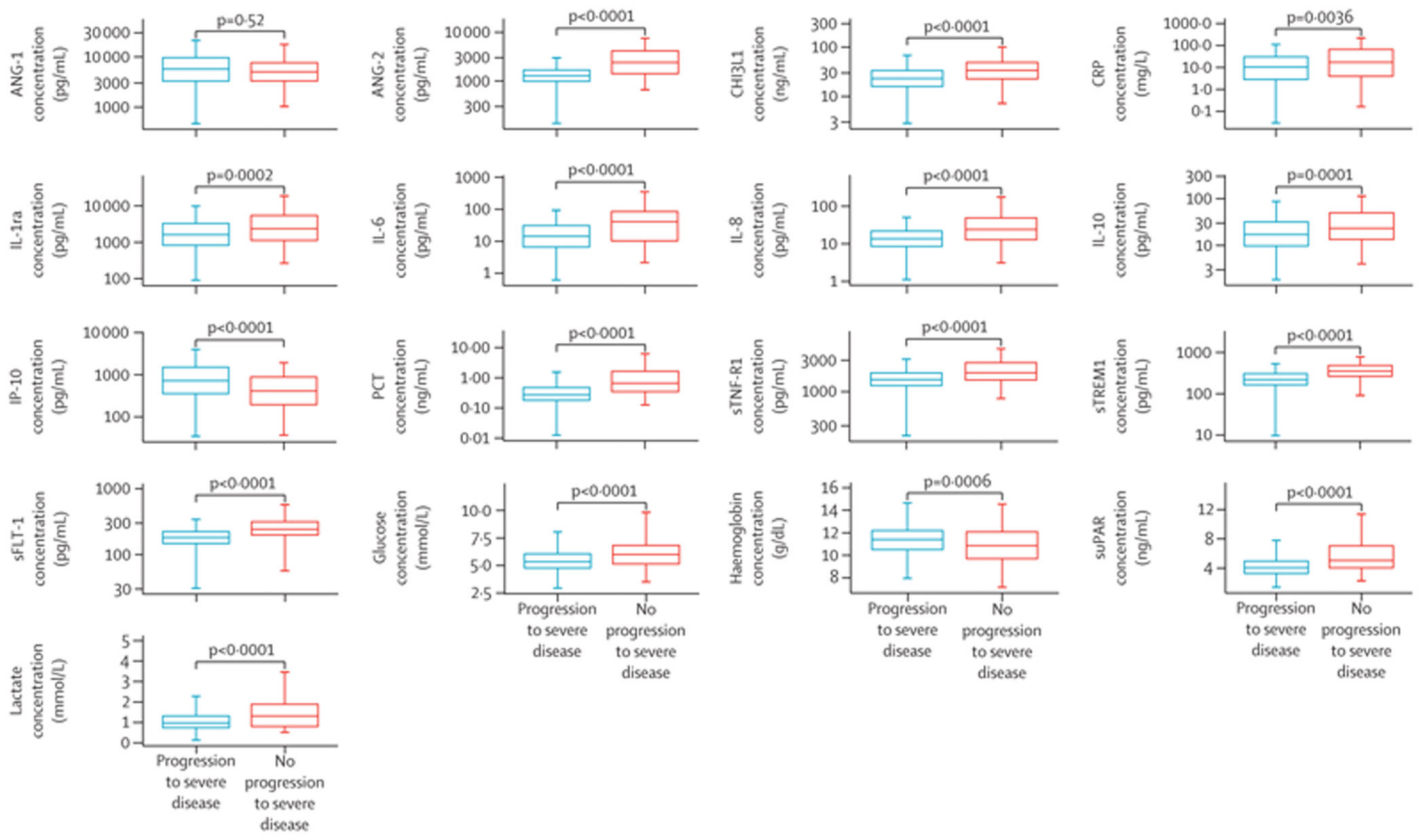
Presenting plasma concentrations of endothelial and immune activation markers, stratified by outcome status Box denotes middle 50% of the data, with the median indicated by the solid horizontal line. Upper hinges denote 75th percentiles and lower hinges denote 25th percentile. Whiskers extend from minimum (lower hinge – 1·5 × IQR) to maximum value (upper hinge + 1·5 × IQR). Outliers were not plotted to aid clarity. s=soluble.

Overall, 133 (3·9%) of 3405 participants met the outcome (39 in Bangladesh, 36 in Cambodia, 32 in one Viet Nam site, and 26 in a second Viet Nam site). 22 participants died, and 111 participants required organ support. The weighted outcome prevalence was 0·34% (95% CI 0·28–0·41; appendix 3 pp 11–12). Young age, age-adjusted tachycardia, abnormal mental state, and bedside signs of poor peripheral perfusion and respiratory compromise at enrolment were more common in participants who progressed to severe disease (table 1). Further participant characteristics, stratified by whether a child progressed to develop severe disease and by site, and characteristics of care received by participants in the community before presenting to the study sites are shown in appendix 3 (pp 13–20). Presence of a WHO danger sign at enrolment was associated with meeting the outcome, as were higher LqSOFA and SIRS scores (table 1). Presenting plasma concentrations of endothelial and immune activation markers, stratified by outcome status, are shown in figure 2 (absolute concentrations are provided in appendix 3 p 15).

**Figure 3:**
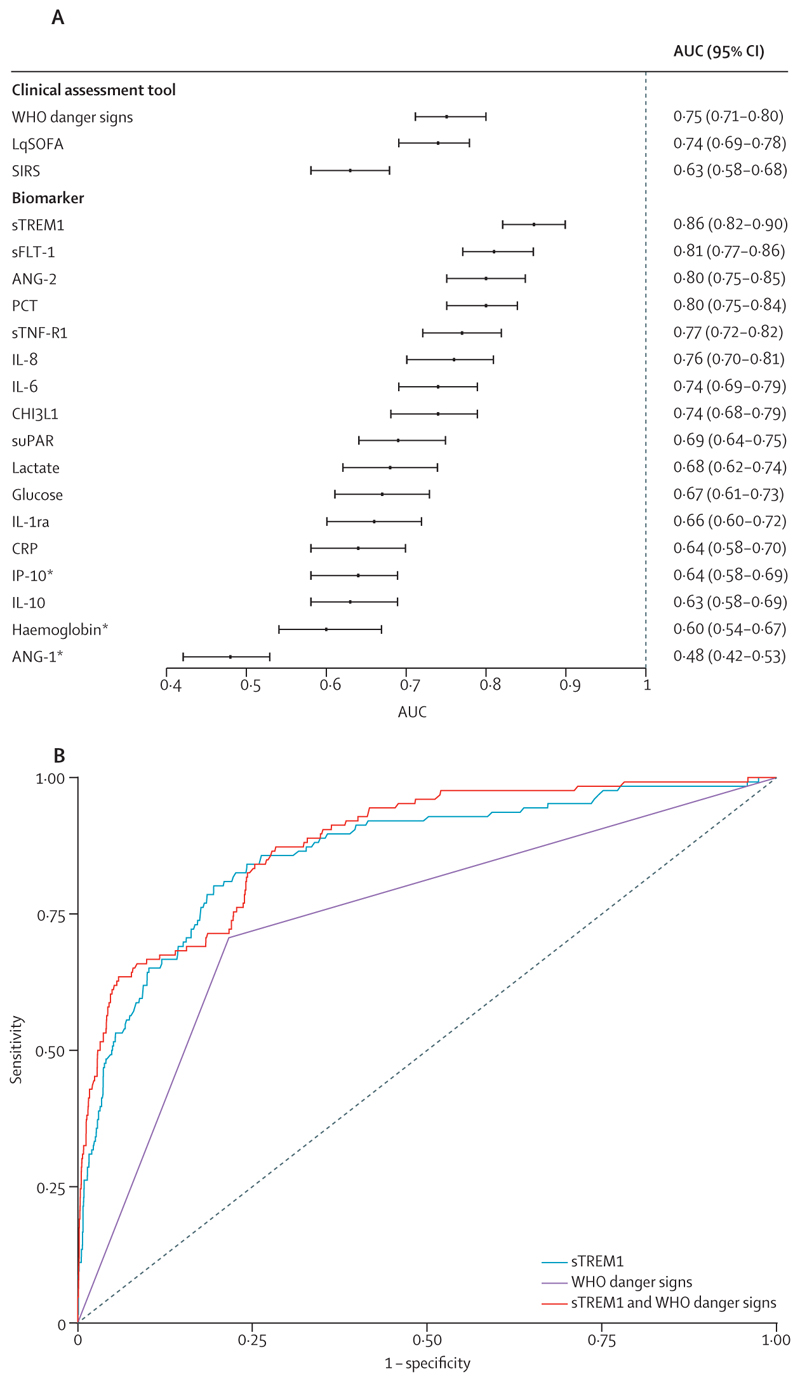
Prognostic performance of biomarkers, WHO danger signs, and clinical severity scores (A) and discrimination for recognising patients who would progress to severe disease of sTREM1, WHO danger signs, and sTREM1 and WHO danger signs combined (B) Error bars indicate 95% CIs. AUC=weighted area under the receiver operating characteristic curve. LqSOFA=Liverpool quick Sequential Organ Failure Assessment. s=soluble. SIRS=systemic inflammatory response syndrome. *Reciprocal concentration used, as lower biomarker concentrations known to be indicative of more severe disease. The ANG-2:ANG-1 ratio was also explored because it might provide a better indication of the balance between microvascular stability and leak: AUC 0·69 (95% CI 0·64–0·75).

sTREM1 showed best prognostic accuracy to discriminate patients who would progress to severe febrile illness (AUC 0·86 [95% CI 0·82–0·90]), compared with other circulating markers, WHO danger signs (0·75 [0·71–0·80]; p<0·0001), and the clinical severity scores (LqSOFA: 0·74 [0·69–0·78]; p<0·0001; SIRS: 0·63 [0·58–0·68]; p<0·0001; figure 3A). Combining WHO danger signs with sTREM1 (0·88 [0·85–0·91]) did not improve performance over sTREM1 alone (p=0·24; figure 3B).

Sensitivity and specificity of WHO danger signs for recognition of children at risk of progression to severe disease was 0·72 (95% CI 0·66–0·79) and 0·79 (0·76–0·82), respectively. sTREM1 concentrations selected to provide equivalent sensitivity (279 pg/mL) or specificity (257 pg/mL) improved classification (table 2). Using the Youden index to identify a sTREM1 threshold for triage (261 pg/mL) resulted in a sensitivity of 0·80 (95% CI 0·73–0·85) and specificity of 0·81 (0·78–0·83). At the current outcome prevalence (0·34%), compared with using WHO danger signs, sTREM1-based triage would identify approximately one additional child who would progress to severe febrile illness for every 3000 patients tested (NNT), without compromising specificity (increasing false positives).

A microbiological cause for infection was confirmed in 898 (26·4%) of 3405 participants: 429 RSV, 164 arboviral infections (109 dengue, 47 chikungunya, and eight Zika virus); 146 influenza (87 influenza A and 59 influenza B); 81 SARS-CoV-2; 59 human metapneumovirus; 19 bacteraemias; nine rickettsial infections (six *Rickettsia* spp and three *Orientia tsutsugamushi*); nine pertussis (eight *Bordetella parapertussis* and one *Bordetella pertussis*); four leptospirosis; three *Chlamydia pneumoniae*; and three *Mycoplasma pneumoniae* (appendix 3 p 21). 34 participants had co-infection with two pathogens. Among participants with microbiologically confirmed infections, prognostic accuracy of the circulating markers, WHO danger signs, and LqSOFA was largely unchanged (appendix 3 p 22), with sTREM1 providing the best discrimination (AUC 0·88 [95% CI 0·83–0·94]). Few participants had confirmed bacterial infections (n=47), precluding comparison of prognostic performance between participants with viral and bacterial infections.

In participants whose presentations met WHO diagnostic criteria for pneumonia (cough or difficulty breathing, with age-adjusted tachypnoea or chest indrawing),^6^ prognostic accuracy of sTREM1 (AUC 0·84 [95% CI 0·78–0·89]; appendix 3 p 22) was matched by ANG-2 (0·85 [0·79–0·91]) and sFLT-1 (0·84 [0·77–0·90]). Prognostic accuracy of clinical assessment tools was inferior (appendix 3 p 22). Similar results were obtained when analyses were restricted to undernourished participants (weight-for-age Z score <–2; appendix 3 p 22).

**Figure 4:**
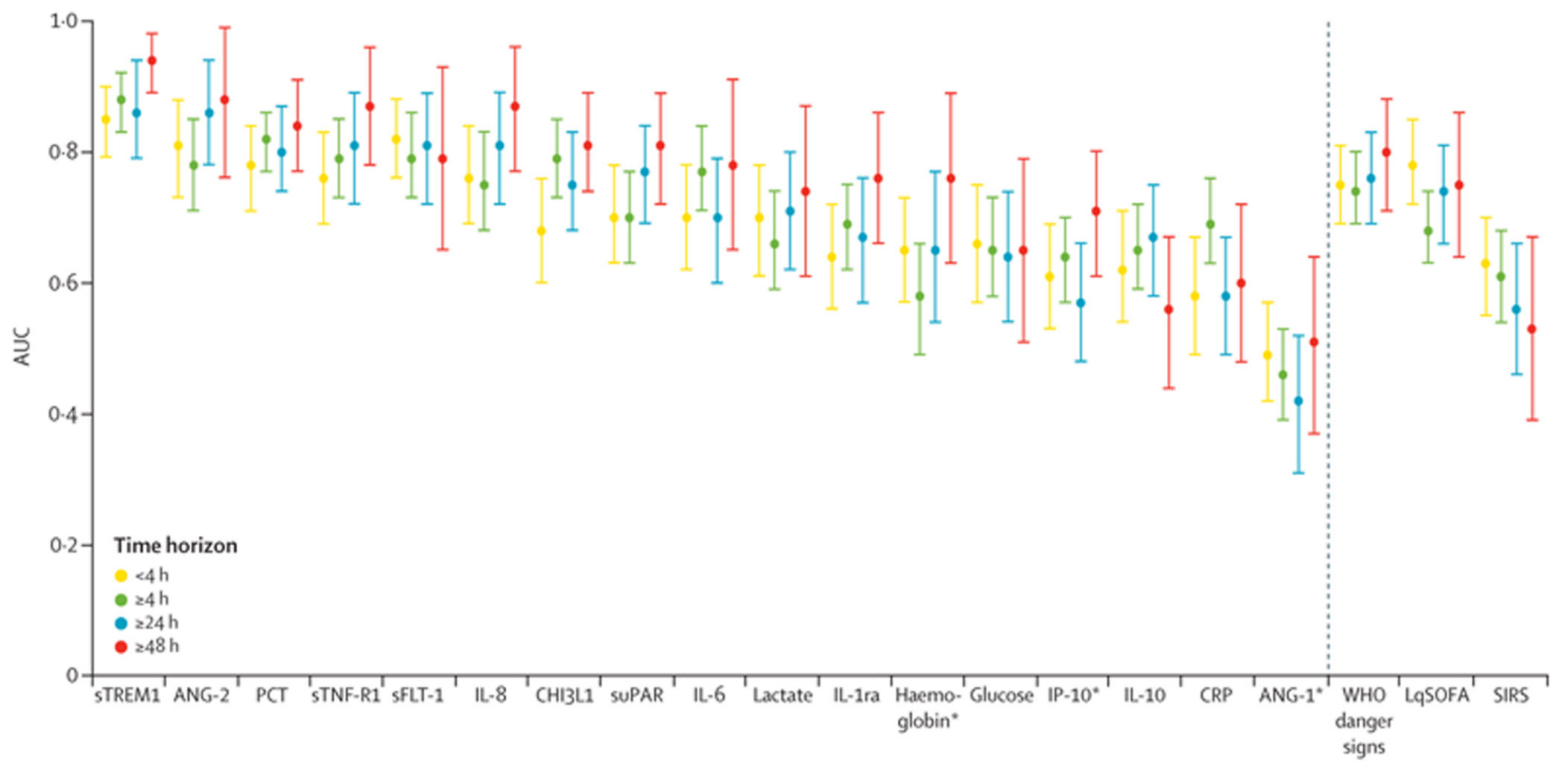
Prognostic accuracy of biomarkers, WHO danger signs, and clinical severity scores across prediction horizons Prediction horizons were <4 h, ≥4 h, ≥24 h, and ≥48 h. Participants who met the outcome before 4 h, 24 h, or 48 h are excluded from the analyses of the ≥4h, ≥24 h, and ≥48 h time horizons, respectively. Biomarkers presented to the left of the dashed vertical line, clinical assessment tools presented to the right, both in descending order of mean AUC across prediction horizons. Circles indicate the point estimate for the AUC. Error bars indicate 95% CIs. AUC=weighted area under the receiver operating characteristic curve. LqSOFA=Liverpool quick Sequential Organ Failure Assessment. s=soluble. SIRS=systemic inflammatory response syndrome. *Reciprocal concentration used, as lower biomarker concentrations are known to be indicative of more severe disease.

Extending the prediction horizon to include all cases of severe febrile illness occurring during follow-up (up to day 28) identified an additional ten participants (two deaths, and eight survivors who required organ support). Data for time-stratified analyses were available for 139 (97·2%) of 143 participants: 56 of 3401 participants met the outcome within 4 h of enrolment, whereas 83 of 3319 after more than 4 h, 42 of 3278 after more than 24 h, and 21 of 3257 after more than 48 h after enrolment (appendix 3 p 23). For most circulating markers, there was improved discrimination at distal prediction horizons (figure 4; appendix 3 pp 24–25), whereas WHO danger signs performed consistently across prediction horizons and clinical severity scores performed better for participants whose illnesses progressed soon after enrolment. sTREM1 out-performed other markers and clinical assessment tools across all prediction horizons, demonstrating an AUC of 0·94 (95% CI 0·89–0·98) for discrimination of participants who progressed to severe disease more than 48 h after presentation.

sTREM1 maintained prognostic accuracy and outperformed the clinical assessment tools across sites (AUCs 0·84–0·89; appendix 3 pp 26–27). Sensitivity analyses excluding the northern Viet Nam site (n=612), which departed from the ideal rural target site profile and where outpatient weighting was derived using different methodology (appendix 3 pp 11–12, 26–27), did not affect the results. Finally, in a sensitivity analysis restricted to participants who had not received parenteral treatment at the study site before baseline data or sample collection (3037 [89·2%] of 3405), sTREM1 remained the best prognostic indicator (AUC 0·82 [95% CI 0·76–0·88]; appendix 3 pp 28–29).

## Discussion

In this large and geographically diverse study investigating circulating markers of immune and endothelial activation for the risk stratification of unselected children with febrile illness presenting from community settings in Asia, we found sTREM1 to be consistently superior to WHO danger signs (the current standard of care) and LqSOFA (a validated clinical severity score) across prediction horizons, sites, presenting clinical syndromes, and in participants with microbiologically confirmed infections.

Previous studies^11,16,17,37^ highlight the promising prognostic performance of sTREM1 but focused exclusively on children admitted to hospital. We have completed, to our knowledge, the first multi-country evaluation at the community level, where the need for better triage tools is most urgent.^1,38^ We identified sites serving as a first point of presentation for rural populations, enrolled unselected patients aged 1–59 months with febrile illness including outpatients, recruited participants immediately upon presentation, excluded patients admitted elsewhere before screening, and adopted an analysis strategy ensuring an outcome prevalence reflective of community care settings.^34,35^ Other key strengths include the inclusion of a prespecified panel of biomarkers compiled based on existing literature and underpinned by mechanistic links to sepsis pathophysiology, which lends face validity to the findings; simultaneous quantification of multiple markers in a central laboratory to ensure comparability of findings; and recruitment across seven sites in five countries for longer than 30 months, which improves geographical and seasonal generalisability.

Our findings lend support to the evidence that certain circulating markers of endothelial and immune activation are pathogen agnostic, reflecting final common pathways to severe infection.^12,23,24^ Thus, they are attractive candidates for risk stratification in primary care, where the cause of infection is typically unknown at the time of triage and recognising which child’s illness is likely to progress remains a major clinical challenge. Endothelial dysfunction has been demonstrated in ambulatory children with infection.^39^ However, until now, it was unclear whether concentrations of these markers would be elevated sufficiently early in the natural history of infection to permit their use for risk stratification at the community level. The results of this study, in conjunction with two previously published smaller community-based studies, suggest that this approach warrants further attention.^15,40^

The results of our study are consistent with a study of 507 adults with febrile illness attending outpatient clinics in Tanzania, which reported an AUC for sTREM1 of 0·87 (95% CI 0·81–0·92) for predicting death within 28 days.^40^ In a study of children with pneumonia presenting to a primary care clinic on the Thailand–Myanmar border, ANG-2 demonstrated the best prognostic performance (AUC 0·81 [95% CI 0·74–0·87]) to predict supplemental oxygen requirement, whereas sTREM1 did not show discriminatory value (0·56 [0·49–0·63]).^15^ In part, this might relate to the focus on pneumonia: ANG-2 was the top-performing marker among participants with pneumonia in our study, although performance of sTREM1 remained similar. Alternatively, the contrasting findings might be explained by the more proximal endpoint (supplemental oxygen requirement *vs* organ support and death) or pre-analytical differences in sample matrix or storage conditions, which are known to influence biomarker concentrations.^41^

Our outcome measure was selected as it is unlikely to be influenced by factors other than disease severity. Small amounts of outcome misclassification can substantially affect estimates of predictor performance.^42^ Nevertheless, predictors of severe disease might not generalise to more proximal outcomes. Studies of COVID-19 and childhood pneumonia indicate that sTREM1 concentrations do not predict supplemental oxygen requirement as accurately as they do mortality.^15,17,43^ This underscores the importance of including a range of outcomes when evaluating the performance of predictors in primary care.

Several limitations of this study reflect challenges inherent in severity prediction at the community level. Despite steps taken to optimise external validity to community settings, differences between patients presenting to rural hospital outpatient departments and primary care facilities will remain. Among participants progressing to severe febrile illness, median time to developing severe disease was 6 h (IQR 2–30), indicating a level of severity at presentation that might not be replicated in some community care settings. Nevertheless, time-stratified analyses demonstrate that discrimination of most circulating markers was best in patients who progressed to severe disease later; a finding not observed for the comparator clinical assessment tools. Although participants who had met the outcome before a time horizon were excluded for each particular analysis (ie, each analysis was conditional on children surviving to that point), the results suggest that biomarkers might be of greatest value in patients whose illness severity is not clinically apparent at presentation and who are at risk of being sent home and deteriorating. This is consistent with previous work in childhood pneumonia and has implications for operationalising biomarker tests.^15^ Our analyses were structured such that every participant would receive a biomarker test. Although this might be appropriate in certain settings,^1,44^ in others a point-of-care test would probably be used selectively on children for whom decisions to refer are borderline. Future work must explore different strategies for integrating biomarker testing into patient triage and compare the cost-effectiveness and feasibility of different approaches.

In settings in which front-line health workers have limited training and supervision, single-biomarker triage tools might offer meaningful clinical value.^1^ Integrating new biomarker tests into existing systems—such as combining them with malaria rapid diagnostic tests in areas where children with febrile illness are routinely screened—could encourage adoption, enhance patient and provider experience, and build on decades of investment in point-of-care testing capacity among community health-care worker networks.^44,45^ However, biomarker tests are not a substitute for clinical judgement. Research is ongoing to develop and validate clinical prediction models that incorporate biomarkers into multivariable frameworks, which include basic clinical assessments, to better quantify the added value biomarkers bring to clinical decision making. Although we limited our analyses to single biomarkers in this study, it might be that multi-biomarker models or alternative methods (eg, classification and regression tree analyses or machine learning algorithms) might identify biomarker combinations with higher prognostic value. Any gains in accuracy would need to be weighed against the feasibility of measuring multiple biomarkers in resource-constrained community settings.

The impact of the COVID-19 pandemic must be considered. Most participants were tested for SARS-CoV-2 (2861 [84·0%] of 3405), and few were infected (81 [2·8%] of 2861). Findings should not be biased towards biomarkers implicated in this specific infection. Nevertheless, health systems and care-seeking pathways were substantially affected during the pandemic, with both attendance rates and the proportion of patients with severe outcomes generally lower than anticipated based on pre-pandemic baseline data. In particular, no severe outcomes were observed at the Indonesia or Laos sites. It will be important to assess the generalisability of our results in non-pandemic times.

Concentrations of circulating markers of immune and endothelial activation predict disease severity across a spectrum of common childhood infections. We demonstrate that these findings might be applicable at the community level, where need for better risk stratification tools is most urgent. Among the markers studied, sTREM1 holds most potential for clinical and public health impact, demonstrating ability to identify one additional child progressing to life-threatening infection for every approximately 3000 children tested, compared with the existing standard of care. Future work should focus on validating these findings and explore different approaches for integrating biomarker testing into patient triage that consider accuracy, costeffectiveness, and patient–provider workflow. Priority should be given to biomarkers that are harbingers for disease progression and facilitate earlier recognition of patients in whom illness severity is not yet clinically apparent at presentation. Ultimately, point-of-care tests for the most promising biomarkers must be developed if the clinical utility of biomarker-based triage strategies is to be assessed in definitive randomised controlled trials.

## Supplementary Material

Supplementary material

## Figures and Tables

**Table 1:** Presenting clinical characteristics, stratified by disease outcome

	Overall (n=3405)	Non-severe disease (n=3272)	Severe disease (n=133)	p value*
Demographics and clinical history				
Age, months	16·8 (8·7 to 31·0)	17·3 (9·1 to 31·3)	4·9 (2·6 to 17·3)	<0·0001
Sex				
Male	2029 (59·6%)	1941 (59·3%)	88 (66·2%)	0·11
Female	1376 (40·4%)	1331 (40·7%)	45 (33·8%)	··
Known comorbidity	102 (3·0%)	100 (3·1%)	2 (1·5%)	0·44
Overnight admission in the past 6 months	429/3394 (12·6%)	412/3262 (12·6%)	17/132 (12·9%)	0·93
Anthropometrics				
Weight-for-age Z score	–0·8 (–1·7 to 0·0)	–0·8 (–1·7 to 0·1)	–1·1 (–2·5 to –0·2)	0·0036
Wasted (weight-for-height Z score <–2) †	585/3393 (17·2%)	551/3261 (16·9%)	34/132 (25·8%)	0·0082
Stunted (height-for-age Z score <–2)	664/3401 (19·5%)	629/3268 (19·2%)	35 (26·3%)	0·044
Illness history				
Duration of illness, days	3·0 (2·0 to 4·0)	3·0 (2·0 to 4·0)	3·0 (2·0 to 5·0)	0·0026
Sought care before presentation	1753 (51·5%)	1681 (51·4%)	72 (54·1%)	0·53
Travel time to study site ≤1 h	2777 (81·6%)	2682 (82·0%)	95 (71·4%)	0·0021
Presenting syndrome				
Upper respiratory tract infection	1121 (32·9%)	1082 (33·1%)	39 (29·3%)	0·37
Lower respiratory tract infection	1347 (39·6%)	1261 (38·5%)	86 (64·7%)	<0·0001
Diarrhoeal	646 (19·0%)	631 (19·3%)	15 (11·3%)	0·021
Neurological	430 (12·6%)	416 (12·7%)	14 (10·5%)	0·46
No focus	527 (15·5%)	519 (15·9%)	8 (6·0%)	0·0021
Severity at presentation				
Any WHO danger sign present	1607/3398 (47·3%)	1512/3266 (46·3%)	95/132 (72·0%)	<0·0001
Prostration	240 (7·0%)	192 (5·9%)	48 (36·1%)	<0·0001
Intractable vomiting	687/3401 (20·2%)	645/3268 (19·7%)	42 (31·6%)	0·0009
Convulsions	433/3400 (12·7%)	419/3268 (12·8%)	14/132 (10·6%)	0·45
Lethargy	825/3399 (24·3%)	764/3267 (23·4%)	61/132 (46·2%)	<0·0001
LqSOFA score	··	··	··	<0·0001
0	2639/3402 (77·6%)	2579/3269 (78·9%)	60 (45·1%)	··
1	642/3402 (18·9%)	596/3269 (18·2%)	46 (34·6%)	··
2	98/3402 (2·9%)	78/3269 (2·4%)	20 (15·0%)	··
3	20/3402 (0·6%)	15/3269 (0·5%)	5 (3·8%)	··
4	3/3402 (>0·1%)	1/3269 (>0·1%)	2 (1·5%)	··
Median (IQR)	0 (0 to 0)	0 (0 to 0)	1 (0 to 1)	<0·0001
SIRS score‡	··	··	··	0·0039
0	223/2827 (7·9%)	222/2707 (8·2%)	1/120 (0·8%)	··
1	1042/2827 (36·9%)	998/2707 (36·9%)	44/120 (36·7%)	··
2	916/2827 (32·4%)	878/2707 (32·4%)	38/120 (31·7%)	··
3	495/2827 (17·5%)	471/2707 (17·4%)	24/120 (20·0%)	··
4	151/2827 (5·3%)	138/2707 (5·1%)	13/120 (10·8%)	··
Median (IQR)	2 (1 to 2)	2 (1 to 2)	2 (1 to 3)	0·0064
Vital signs				
Heart rate, bpm§				
Age 1 month to <12 months	156·0 (140·0 to 172·0)	155·0 (140·0 to 170·0)	171·5 (157·5 to 186·0)	<0·0001
Age 12 months to <60 months	140·0 (127·0 to 158·0)	140·0 (126·0 to 157·0)	160·0 (140·0 to 177·0)	<0·0001
Respiratory rate, breaths per min¶				
Age 1 month to <12 months	45·0 (38·0 to 55·0)	44·0 (37·0 to 53·0)	59·5 (48·0 to 66·0)	<0·0001
Age 12 months to <60 months	36·0 (30·0 to 42·0)	36·0 (30·0 to 42·0)	39·0 (32·0 to 55·0)	0·0055
Oxygen saturation, %‖	98·0 (97·0 to 99·0)	98·0 (97·0 to 99·0)	97·0 (95·0 to 98·0)	<0·0001
Axillary temperature, °C**	37·6 (37·0 to 38·3)	37·6 (37·0 to 38·3)	37·6 (36·9 to 38·3)	0·98
Capillary refill time >2 s	194 (5·7%)	165 (5·0%)	29 (21·8%)	<0·0001
Not alert	86 (2·5%)	67 (2·0%)	19 (14·3%)	<0·0001

Data are median (IQR), n (%), or n/N (%). LqSOFA=Liverpool quick Sequential Organ Failure Assessment. SIRS=systemic inflammatory response syndrome.

* p values were calculated using the Wilcoxon rank sum test, Pearson’s χ^2^ test, or Fisher’s exact test.

† Calculated in children with a height of 45–120 cm.

‡ Missingness for SIRS was high because leukocyte count is required and complete blood counts were measured at the discretion of the treating clinical team.

§ Data missing for one participant with nonsevere illness.

¶ Data missing for two participants with non-severe illness.

|| Data missing for 205 participants, 145 with non-severe illness and 60 with severe illness.

** Data missing for one participant with non-severe illness.

**Table 2:** Sensitivity and specificity of WHO danger signs and sTREM1 for recognition of participants who progressed to severe disease

	Threshold	Method for selection	Sensitivity (95% CI)	Specificity (95% CI)
WHO danger signs	Presence of at least one sign	Not applicable	0’72 (0-66-0-79)	0.79 (0.76-0.82)
sTREMl	279 pg/mL	Fixed at sensitivity of WHO danger signs	0-72 (0-65-0-79)	0.83 (0.81-0.86)
sTREMl	257 pg/mL	Fixed at specificity of WHO danger signs	0.80 (0-74-0-86)	0.79 (0.76-0.82)
sTREMl	261 pg/mL	Youden index	0’80 (0.73-0.85)	0.81 (0.78-0.83)

sTREM1=soluble TREM1.

## Data Availability

De-identified, individual participant data from this study will be available to researchers whose proposed purpose of use is approved by the data access committees at Médecins Sans Frontières and the Mahidol Oxford Tropical Medicine Research Unit (MORU). Enquiries or requests for data can be sent to data.sharing@london.msf.org and datasharing@tropmedres.ac. Researchers interested in accessing biobanked samples should contact the corresponding author, who will coordinate with the Spot Sepsis Sample Use Committee.

## Research in context

### Evidence before this study

We searched MEDLINE, Embase, and Web of Science from May 31, 1999, to April 8, 2023, without language restrictions, using synonyms of “fever”, “paediatric”, “prediction”, and “severe infection” (appendix 3 pp 2–3). Studies in both high-income and low-income settings show that circulating markers of immune and endothelial activation can be used to stratify risk of poor outcomes in common childhood infections, including pneumonia, meningitis, malaria, and dengue. However, most studies to date have been single-centre and pathogen-specific, recruiting patients partway through their hospital stay in settings with high prevalence of severe disease, limiting generalisability to community settings where patients present early in their illness and where the cause of infection is typically unknown at the time of presentation.

### Added value of this study

For children presenting from the community with febrile illness, soluble TREM1 (sTREM1) consistently outperformed other circulating markers of immune and endothelial activation and WHO danger signs (standard of care) in predicting progression to severe febrile illness. Prognostic accuracy was consistent across sites, prediction horizons, and presenting syndromes, and in microbiologically confirmed infections. Prognostic accuracy was greatest among participants who deteriorated more than 48 h after enrolment. This finding, together with the notable improvement in sensitivity compared with WHO danger signs, suggests that biomarker measurements could provide an additional diagnostic safety layer for patients whose illness severity is not apparent at presentation.

### Implications of all the available evidence

A recent global stakeholder consultation concluded that prognostic tools for febrile illnesses are urgently required in resource-constrained community contexts. This multi-country study provides the first large-scale evaluation of circulating markers of immune and endothelial activation for risk stratification of childhood infections for patients presenting from community contexts in Asia. Future research should explore implementation and cost-effectiveness of strategies for integrating biomarker testing into patient triage. Point-of-care tests are required before clinical utility of sTREM1-based triage strategies can be evaluated in randomised controlled trials.
